# Case Report: Deep sternal wound infection caused by *Mycoplasma hominis* after cardiac surgery

**DOI:** 10.3389/fcvm.2025.1538389

**Published:** 2025-05-12

**Authors:** Zhaohui Wang, Yong Shi, Yu Chen, Lijun Chen

**Affiliations:** ^1^ Department of Department of Cardiac Surgery, Affiliated Dong Yang Hospital of Wenzhou Medical University, Dong Yang, Zhejiang, China; ^2^ Department of Ultrasonography, Affiliated Dong Yang Hospital of Wenzhou Medical University, Dong Yang, Zhejiang, China

**Keywords:** *Mycoplasma hominis*, deep mediastinitis, cardiac surgery, omadacycline, DSWI

## Abstract

Deep sternal wound infection (DSWI) caused by *Mycoplasma hominis* after cardiac surgery is very rare and easily overlooked. This paper reports the case of a patient with DSWI after cardiac surgery. The patient had an unexplained fever postoperatively. On the seventh day after surgery, the incision opened, and there was pus leakage. On the 11th day after surgery, the pus culture indicated *Mycoplasma hominis* infection. The patient was cured after treatment with omadacycline and secondary surgery.

## Introduction

Deep sternal wound infection (DSWI) is one of the most serious complications after cardiac surgery through the sternum, with an incidence rate worldwide ranging from 0.5% to 5.6% (1). Patient risk factors include female gender, age, diabetes, renal failure, smoking, obesity, breast size, steroid use, and chronic obstructive pulmonary disease (2, 3). Surgery-related risk factors include surgery duration, internal mammary artery use, re-thoracotomy, blood product use, intensive care unit stay, and duration of mechanical ventilation (4, 5). The most common pathogens are Methicillin-sensitive *Staphylococcus aureus* (45%), Methicillin-resistant *S. aureus* (16%), Gram-negative bacilli (17%), Coagulase-negative Staphylococci (13%), and Streptococci (5%) (6). *Mycoplasma hominis* is a rare pathogen for DSWI, with less than 20 cases of postcardiac surgery *M. hominis* mediastinitis reported globally in the past 50 years (6, 7). This paper reports a case of a patient who underwent valvular replacement and coronary artery bypass grafting and presented with fever and chest pain postoperatively. *M. hominis* was found in the incision pus culture. The patient showed improvement after standardized anti-infection treatment and pectoralis major muscle flap transfer and was discharged in good condition.

## Case presentation

A 59-year-old man was admitted to the hospital for “chest tightness and shortness of breath after exertion for 1 month.” He had a history of “cerebral infarction” and underwent coronary stent implantation 1 year ago at a hospital in another underdeveloped province about 2,000 km far away. He came to our hospital for medical treatment through a health assistance project between the two provincial governments. He denied any other medical history. Echocardiography in our hospital revealed moderate stenosis with moderate insufficiency of the aortic valve. The ejection fraction (EF)% value was 68%. Coronary angiography indicated 85% stenosis in the middle of the right coronary artery and 80%–90% stenosis in the distal segment of the left circumflex branch. The patient's predicted mortality was 1.29% based on EuroSCORE II. The calculated additive EuroSCORE II value was 4.333.

The patient underwent mechanical aortic valve replacement combined with coronary artery bypass grafting on 26 September 2024. Two graft procedures were performed: a saphenous vein sequential graft, harvested from the right thigh, was anastomosed from the ascending aorta to the left circumflex coronary artery and subsequently to the posterior descending branch of the right coronary artery.

The total surgical time was 380 min, of which the cardiopulmonary bypass time was 127 min. The aortic cross-clamp time during surgery was 180 min.

After the patient was admitted to the surgical intensive care unit (SICU) after surgery, the SICU nurse reported that he had a swelling of the foreskin and several small ruptures in the local area (unfortunately, the photographs taken at that time are missing now). Local disinfection and foreskin reduction were immediately performed, and the swelling gradually reduced.

**Figure 1:**
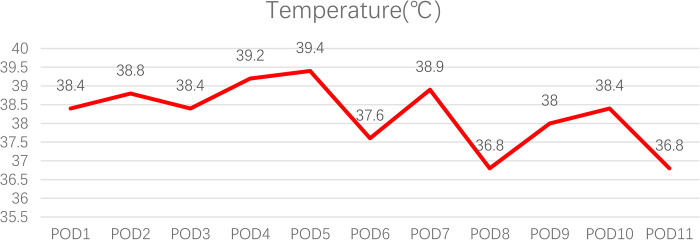
Postoperative changes in temperature.

**Figure 2:**
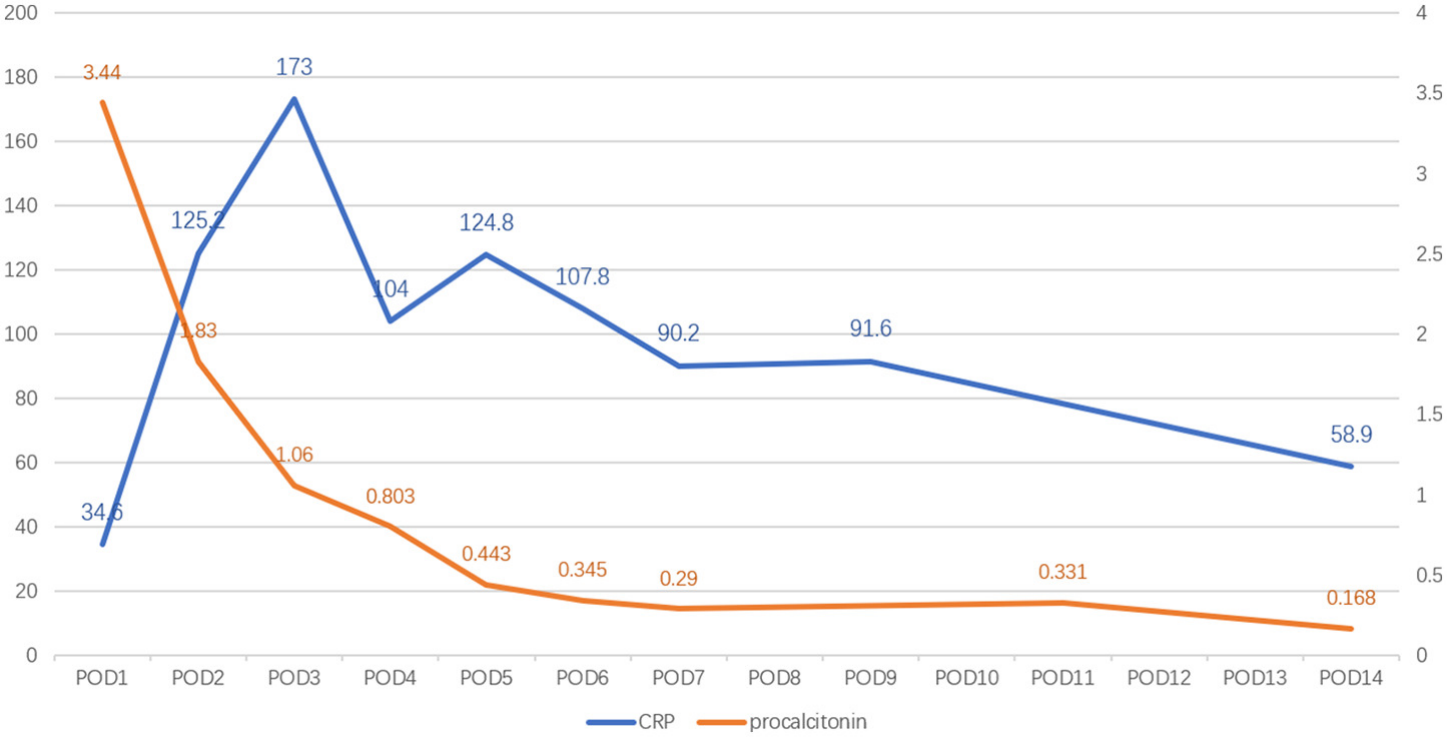
Postoperative changes in CRP (mg/L) and procalcitonin (ng/ml).

The patient had a fever on postoperation day (POD) 1, with the highest temperature reaching 39.4°C on POD 5. It was not until POD 11 that the body temperature returned to normal (Figure 1). The patient's white blood cell count and the percentage of neutrophils were slightly elevated. Procalcitonin levels decreased over time, which was considered to have no guiding significance for *M. hominis* infection. C-reactive protein (CRP) levels changed with time and treatment measures (Figure 2).

The urinary catheter was removed on POD 7, and the central venous catheter was removed on POD 8. Chest CT scans performed on POD 1 revealed mild consolidation in the bilateral lower lung lobes and minimal pleural effusion, with subsequent imaging on POD 6 demonstrating improvement in these findings; no mediastinal abnormalities were detected in either scan.

**Figure 3:**
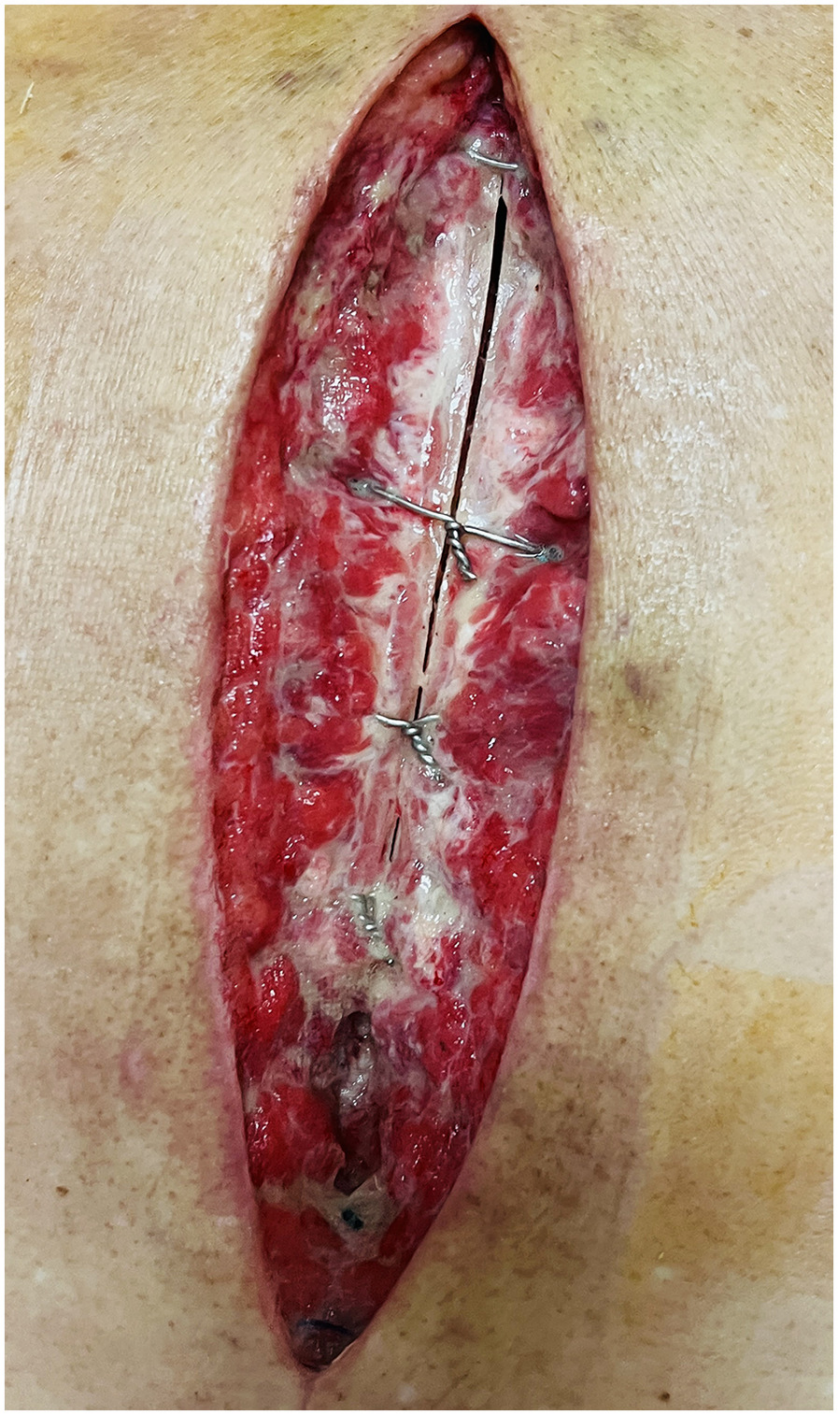
Incision dehiscence.

On POD 7 (3 October 2024), the patient's incision was dehisced, revealing a large amount of yellow-white pus exuding, with poor healing of subcutaneous tissue and sternum (Figure 3).

Before sternal dehiscence, localized erythema and tenderness around the incision were observed on POD 3. However, these signs were initially attributed to routine postoperative inflammation without special attention.

**Figure 4:**
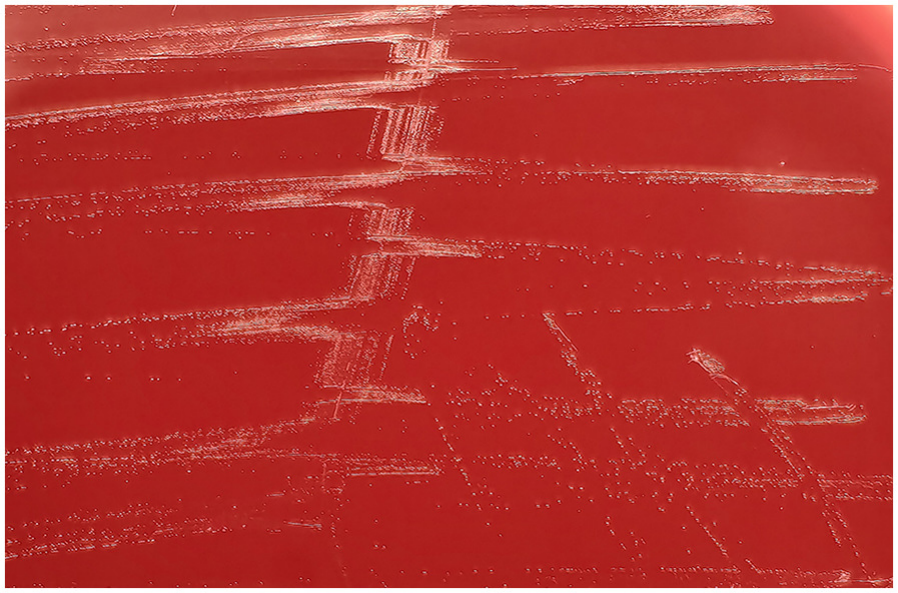
Cultivation results.

Multiple continuous samplings were taken from the incision for culture since sternal dehiscence. Finally, on POD 11, the first positive culture result came out, which indicated *M. hominis* (Figure 4). An antibiogram was not performed due to technical limitations in *M. hominis* susceptibility testing, which is rarely conducted in clinical practice, and the absence of institutional infrastructure for such specialized assays. After a multidisciplinary team discussion, Omadacycline was administered for anti-infection treatment. After using the medication, the incision condition gradually improved, and no positive results were cultured again. On POD 21, an open chest debridement and bilateral pectoralis major muscle flap plugging surgery was successfully performed. The patient was discharged 14 days after the secondary surgery.

The anti-infection treatment is detailed in Table 1.

**Table 1:** Anti-infection treatment detail.

Mediation	Dosage (g)	Frequency of use	Start time	End time
Cefuroxime	1.5	Every 8 h	26 September 2024 16:18	28 September 2024 09:33
Piperacillin–tazobactam	4.5	Every 8 h	28 September 2024 09:33	30 September 2024 08:50
Meropenem	1	Every 8 h	30 September 2024 08:50	2 October 2024 10:50
Piperacillin–tazobactam	4.5	Every 8 h	3 October 2024 09:22	17 October 2024 09:07
Omadacycline	0.1	Everyday	7 October 2024 16:38	29 October 2024 09:22
Meropenem	1	Every 8 h	17 October 2024 18:03	25 October 2024 09:10
Vancomycin	1	Every 12 h	17 October 2024 18:03	25 October 2024 09:10

At 1-, 3-, and 5-month follow-ups, the patient exhibited no fever recurrence, with complete sternal wound healing confirmed on physical examination. Cardiac function remained normal, as evidenced by transthoracic echocardiography and electrocardiography. In chest CT scans, no mediastinal abnormalities were observed. Laboratory assessments revealed normalized inflammatory markers and stable coagulation profiles (international normalized ratio: 1.8–2.5 under warfarin therapy). The patient resumed full-time work and daily activities without functional limitations.

The patient initially expressed frustration due to incision dehiscence and delayed diagnosis. Through detailed communication and empathic counseling, his understanding and adherence improved. At discharge, he reported high satisfaction with the care team's efforts and gratitude for the successful outcome.

## Discussion

DSWI is a rare but potentially devastating complication of median sternotomy performed in cardiac surgery. Risk factors can be broadly divided into preoperative, intraoperative, and postoperative factors, including female sex, obesity, diabetes mellitus, smoking and chronic obstructive pulmonary disease (COPD), bilateral internal mammary artery harvesting, prolonged cardiopulmonary bypass time, and re-exploration for bleeding. In this case, the patient exhibited the following DSWI risks: smoking and single internal mammary artery harvesting. The risk factors had limited guiding significance for this patient.

*M. hominis* infection causing DSWI after cardiac surgery is very rare and difficult to diagnose. The main reason is that *M. hominis* has high requirements for culture, and ordinary culture methods make it difficult to obtain positive results (8). Currently, many studies have adopted molecular diagnosis and genomic sequencing methods to diagnose *Mycoplasma* infections (6, 7) and have achieved good results, but new technology means high costs and low popularity. So, conventional culture and testing remain the main methods for grassroots hospitals to find the causative bacteria of infection. First, qualified test specimens and multiple tests may increase the positive detection rate. Second, culture dishes with no bacterial growth within 48 h should not be directly reported as negative and discarded. Previous studies have proven that *M. hominis* requires at least 4 days or even longer under conventional culture to appear in colonies (9).

Many articles also mention an interesting phenomenon about *M. hominis*, which is that all patients with DSWI caused by postoperative *M. hominis* are male. *M. hominis* mainly colonizes in the human respiratory and urinary tract and, because of the anatomical differences between male and female urethras, the damage to the male urethra from catheterization is much greater than that to the female urethra. Therefore, many researchers believe that *Mycoplasma* infection is related to urethral injury. In this case, the patient did indeed have penile edema and local skin ulceration on the first and second days after surgery. In addition, the patient's graft vessel for the bypass was taken from the great saphenous vein of the right thigh, which is close to the perineal area. It is possible that strict aseptic operation was not maintained, leading to *Mycoplasma* infection. Therefore, perioperative perineal cleaning, preoperative catheterization operations, and strict aseptic operations during surgery are crucial for preventing infection.

In this case, the patient was treated with piperacillin–sulbactam and meropenem without therapeutic effect before the pathogen was identified. After the culture confirmed a *Mycoplasma* infection, omadacycline was used for anti-infection treatment. The decision to initiate Omadacycline was guided by a multidisciplinary team consultation involving infectious disease and emergency medicine specialists. Key factors included the following: confirmed *M. hominis* infection with suspected genitourinary origin, given the patient's postoperative penile edema and proximity of the saphenous vein graft harvest site to the perineal region; *in vitro* evidence demonstrating the potent activity of Omadacycline against *Mycoplasma genitalium* [minimal inhibit concentration (MIC) ≤0.5 mg/ml], even in isolates resistant to other tetracyclines (10); and pharmacokinetic advantages, including intravenous administration for rapid therapeutic effect, and broad-spectrum coverage against Gram-positive and Gram-negative pathogens, which mitigated the risks of polymicrobial infection.

Omadacycline is a tetracycline-class antibiotic that inhibits bacterial protein synthesis by binding to the 30S ribosomal subunit. It has antibacterial activity against aerobic Gram-positive bacteria such as *Enterococcus faecalis*, *Enterococcus faecium*, vancomycin-resistant enterococci, methicillin-resistant *S. aureus*, various streptococci, *Corynebacterium jeikeium*, and Enterobacteriaceae resistant to ceftazidime and carbapenems, as well as atypical pathogens (11). In previous case reports on anti-infection treatment with quinolones combined with doxycycline, with or without decortication surgery, 28.6% of patients died due to uncontrollable infection (6).

This case highlights the critical role of persistent culturing with prolonged incubation to isolate *M. hominis*, a fastidious pathogen rarely detected by conventional methods. To our knowledge, it is the first reported use of omadacycline for successfully treating DSWI caused by *M. hominis* after cardiac surgery, addressing a literature gap. Limitations include the single-center, single-case design and the inability to perform susceptibility testing due to institutional constraints, underscoring challenges in managing atypical infections in resource-limited settings.

Prospective multicenter studies with larger cohorts are warranted to validate the efficacy of omadacycline in DSWI caused by *M. hominis*, while advancing rapid molecular diagnostics and standardized susceptibility protocols could enhance the management of such rare, challenging infections.

## Summary

*M. hominis*-induced deep sternal wound infection is a relatively rare complication after cardiac surgery, which is considered to be related to catheterization. *M. hominis* is difficult to culture and, therefore, molecular testing or genomic sequencing methods can be used to assist in diagnosis for units with the capability. For confirmed *M. hominis* infections in DSWI, omadacycline can be used for anti-infection treatment.

## Data Availability

The raw data supporting the conclusions of this article will be made available by the authors without undue reservation.
